# Heat sink effects in thyroid bipolar radiofrequency ablation: an ex vivo study

**DOI:** 10.1038/s41598-023-45926-2

**Published:** 2023-11-07

**Authors:** Konrad Klimek, Nicolai Mader, Christian Happel, Amir Sabet, Frank Grünwald, Daniel Groener

**Affiliations:** Department of Nuclear Medicine, University Hospital Frankfurt, Goethe University, Theodor Stern Kai 7, 60590 Frankfurt, Germany

**Keywords:** Thyroid diseases, Thyroid cancer

## Abstract

The study aimed to investigate heat sink effects in radiofrequency ablation (RFA) under thyroid-specific conditions. In an ex vivo model, bovine thyroid lobes were ablated using bipolar RFA with 2.0 kJ energy input at a power level set to 10 W (n = 35) and 25 W (n = 35). Glass vessels (3.0 mm outer diameter) placed within the ablation zone were used to deliver tissue perfusion at various flow rates (0, 0.25, 0.5, 1, 5, 10, 20 ml/min). Temperature was measured in the proximity of the vessel (T_v_) and in the non-perfused contralateral region of the ablation zone (T_c_), at equal distances to the ablation electrode (d = 8 mm). Maximum temperature within the perfused zone was significantly lowered with T_v_ ranging from 54.1 ± 1.5 °C (20 ml/min) to 56.9 ± 1.5 °C (0.25 ml/min), compared to T_c_ from 63.2 ± 3.5 °C (20 ml/min) to 63.2 ± 2.6 °C (0.25 ml/min) (10 W group). The cross-sectional ablation zone area decreased with increasing flow rates from 184 ± 12 mm^2^ (0 ml/min) to 141 ± 20 mm^2^ (20 ml/min) at 10 W, and from 207 ± 22 mm^2^ (0 ml/min) to 158 ± 31 mm^2^ (20 ml/min) in the 25 W group. Significant heat sink effects were observed under thyroid-specific conditions even at flow rates ≤ 1 ml/min. In thyroid nodules with prominent vasculature, heat dissipation through perfusion may therefore result in clinically relevant limitations to ablation efficacy.

## Introduction

Ultrasound-guided radiofrequency ablation (RFA) has been increasingly adopted for the treatment of thyroid nodules and is now represented in multiple guidelines and consensus statements^1–6^. RFA has proven effective in treating benign symptomatic thyroid lesions through volume reduction and in reverting small autonomously functioning thyroid nodules (AFTN) to euthyroidism^7^. Recent studies have also advocated the application of RFA in early stage differentiated thyroid cancer and selected cases of inoperable disease recurrence^8,9^.

In RFA, heat is generated by a high frequency alternating current applied to the target tissue through an ablation electrode^10^. Thermal energy then disperses in the adjacent tissue by conduction. In the viable tissue, irreversible cell damage is expected to occur instantly at temperatures above 60 °C leaving behind a demarcated coagulation zone^11,12^. Various ablation techniques have been introduced to achieve sufficient heat distribution within thyroid nodules, including the moving shot approach in monopolar RFA and the multi-step overlapping shot technique in bipolar RFA^13–15^.

Perfusion-mediated temperature decrease is known as a potential therapy-limiting phenomenon in thermal treatments. In liver-directed therapies, heat dissipation in the vicinity of large vessels has been shown to have a negative impact on ablation efficacy and clinical outcomes^16,17^. The so-termed heat sink effect has thus been investigated in liver-based ex vivo models for different ablation modalities^18–20^. For thyroid RFA, experimental data is limited, and little is known about the extent of heat sink phenomena under thyroid-specific ablation conditions. As nodule regrowth after thyroid RFA has been observed in long-term follow-up and attributed to the peripheral zone of the treated nodules it has been hypothesized that peripheral hypervascularity plays a significant role in cases of insufficient treatment success^21–23^. Furthermore, when targeting autonomously functioning nodules (AFTN) or differentiated thyroid cancer (DTC), irreversible cell damage within the whole target volume becomes an indispensable treatment goal^8,24^.

This study aims to assess the role of the heat sink effect in bipolar RFA under thyroid-specific ablation conditions. For this purpose, an ex vivo model in bovine thyroid tissue is established. Typical vascular flow rates are applied to the ablation zone, using fixed (10 W) and resistance-modulated power delivery (25 W). Dynamic temperature measurement within the ablation zone and morphometric analysis of cross sections serve as surrogates for ablation zone coverage and treatment efficacy.

## Materials and methods

### Experimental setup

**Figure 1:**
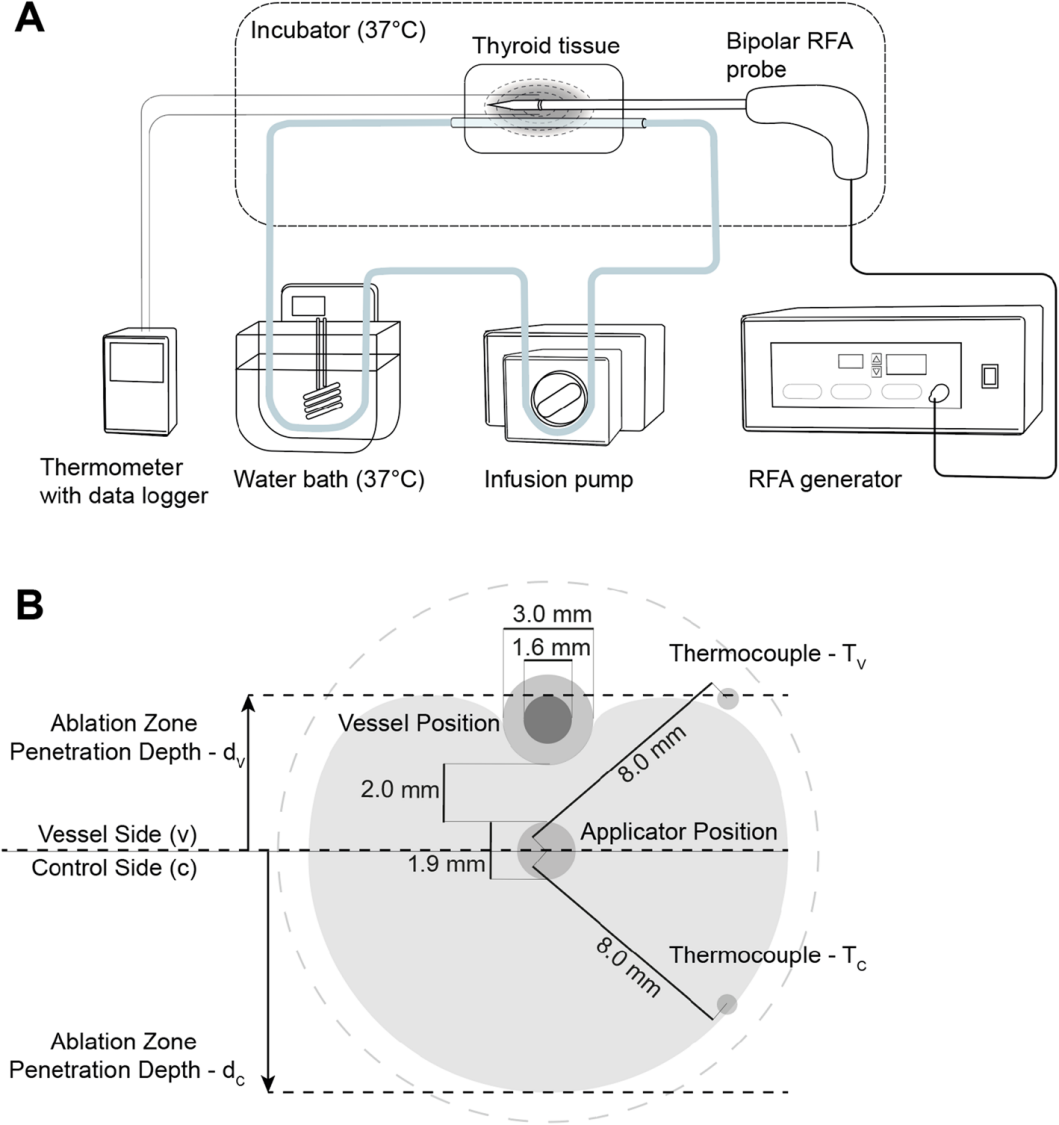
Experimental setup (**A**) for bipolar RFA in ex vivo bovine thyroid samples, maintained at body temperature in an incubator, with variable perfusion rates applied to the ablation zone. (**B**) Cross-section of the ablation zone showing the glass tube position. Equidistant placement of thermocouples in relation to the applicator near the vessel (T_V_) and in the contralateral half of the ablation zone (T_C_).

The study was conducted using bovine thyroid tissue samples. Thyroid glands from freshly slaughtered cows were obtained, the extracted lobes were then placed into a container and maintained at human body temperature (37 °C) in a custom-designed incubator throughout the ablation and subsequent cooling process. A glass tube with an outer diameter of 3.0 mm (inner diameter: 1.6 mm) was positioned in parallel to the ablation probe shaft at a 2-mm distance to mimic an intrathyroidal vessel, as previously described^25,26^. The glass vessel was perfused with water preheated to 37 °C in a water bath (Fig. 1A). Various flow rates were delivered through an infusion pump (Infusomat, B. Braun SE, Melsungen, Germany), including 0, 0.25, 0.5, 1, 5, 10 and 20 ml/min. Thermocouples of a multichannel thermometer (data logger PCE-T390, PCE Deutschland GmbH, Meschede, Germany) were inserted using a custom-made positioning device, allowing for continuous temperature measurement throughout the ablation and cooling period. Based on previous experiments which aimed to identify the 60 °C isotherm contour within the ablation zone cross section (at 10 W input power and 2.0 kJ total energy delivery), thermocouples were placed near the expected outer margin of the coagulation zone at 8 mm distance from the ablation zone center. Maximum temperature was measured in the vicinity of the vessel (T_V_) and at an equidistant position on the contralateral, non-perfused side (T_C_), which served as a control (Fig. 1B).

**Figure 2:**
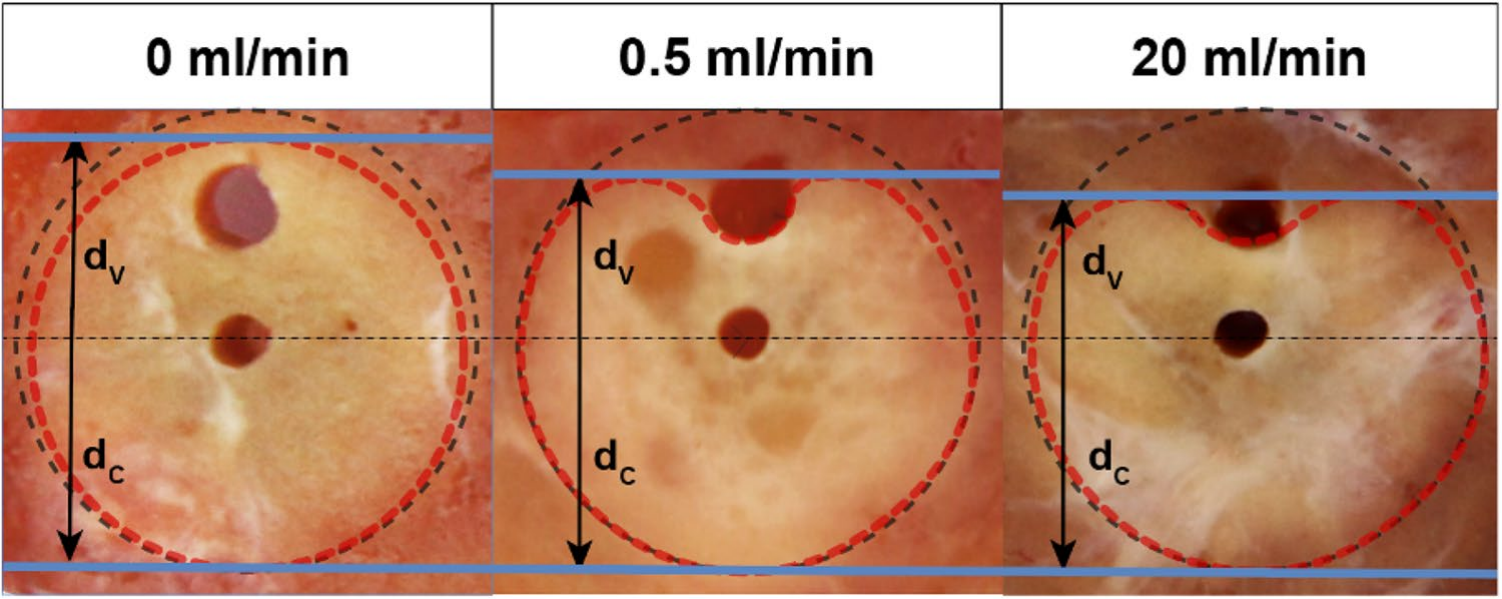
Sample cross-section images of the ablation zone at perfusion rates of 0, 0.5, and 20 ml/min. Measurements of the ablation zone area (red dotted line) and lateral penetration depth (blue line). Decrease of d_V_ with increasing flow rates, while d_C_ remains unchanged.

### Radiofrequency ablation

Ablation was performed with a bipolar radiofrequency ablation system, consisting of a radiofrequency generator (CelonLab POWER, Olympus Surgical Technologies Europe, Hamburg, Germany) and an internally cooled bipolar applicator with a 20 mm active tip and 1.8 mm diameter (CelonProSurge T-20, Olympus Surgical Technologies Europe, Hamburg, Germany). Each ablation was performed on a separate thyroid lobe. The applicator was maintained in a fixed position during the energy application. Power level settings of 10 W and 25 W were chosen based on commonly used pre-sets in clinical practice and to investigate whether the power level setting could influence the extent of potential heat sink effects. To deliver an ablation zone representative of a single shot in bipolar thyroid RFA, a total energy of 2.0 kJ was administered. In the 10 W series, energy was delivered through continuous application of 10 W over 200 s. In the 25 W series, the manufacturer’s adaptive, resistance-controlled automatic power (RCAP) delivery with a maximum input of 25 W was put in place. Five replicate experiments were carried out for each power setting and for each flow rate combination.

### Ablation zone analysis

Central cross-sections of the ablation zone were obtained using a dedicated cutting device (Supplementary Fig. S1) after a 15-min cooling period and photographed (Canon 70D with a 50 mm f1.8 lens, Canon Inc., Japan) next to a millimeter scale. Morphometric data were assessed adopting the methodology set out by Mulier et al.^27^. Quantification of the central “white zone” of coagulation^28^ within the cross-section of the ablated thyroid tissue was carried out using an Excel-based tool (Microsoft Excel Version 2208 (Build 15,601.20578), Microsoft Inc., USA). The total cross-sectional area was quantified, as well as the lateral tissue penetration depth of the coagulation zone in relation to the ablation probe on both the vessel side (d_V_) and the contralateral side (d_c_) (Figs. 1B and 2).

### Statistical analysis

Results are presented as means with standard deviations. For non-parametric comparison of categories, a Mann–Whitney-U-Test was applied, for intraindividual comparison a paired student’s t-test was used, if normal distribution could be assumed. Statistical analyses were performed with GraphPad Prism (version 9.1.1, GraphPad Software, San Diego, CA). All tests were two-sided with *p*-values < 0.05 indicating statistical significance. Within the range of flow rates applied, linear-log regression was used to model the association between the temperatures (T_C_ and T_V_) and the log-transformed vessel flow rate at both power level settings, as well as the relationship between the total ablation zone area and the log-transformed vessel flow rate at both power level settings. An F-test was performed to determine if the slopes significantly differed from zero.

## Results

Radiofrequency ablation was performed in 70 bovine thyroid lobes at power level settings of 10 W (n = 35) and 25 W (n = 35). For each ablation, a total energy of 2.0 kJ was applied. All experiments yielded visually detectable ablation zones in the tissue cross sections. The series with a flow rate of 0 ml/min served as the control group.

### Temperature analysis

Temperatures in the thyroid tissue were measured at equidistant positions from the applicator (*d* = 8 mm) on both the perfused vessel side (T_V_) and the contralateral non-perfused side (T_C_) (Fig. 1). Maximum temperatures T_V_ and T_C_ during the ablation are shown in Table 1.

**Table 1:** Ablation zone area, temperature, and penetration depth at different flow rates and power level settings.

Flow rate (ml/min)	10 W					25 W				
	Area (mm^2^)	T_C_ (°C)	T_V_ (°C)	d_C_ (mm)	d_V_ (mm)	Area (mm^2^)	T_C_ (°C)	T_V_ (°C)	d_C_ (mm)	d_V_ (mm)
0	184.2 ± 11.9	59.6 ± 3.3	58.1 ± 3.4	7.6 ± 0.6	7.1 ± 0.3	206.6 ± 22.4	77.0 ± 5.5	73.7 ± 6.0	8.3 ± 0.7	7.8 ± 0.5
0.25	156.6 ± 25.5	63.2 ± 2.6	56.9 ± 1.5	7.4 ± 0.4	6.5 ± 0.9	205.4 ± 21.6	77.4 ± 9.4	74.6 ± 7.7	8.0 ± 1.0	7.3 ± 0.7
0.5	154.4 ± 17.1	63.4 ± 3.9	56.4 ± 3.0	7.3 ± 1.0	5.6 ± 0.7	195.2 ± 10.1	72.6 ± 12.2	70.2 ± 14.7	8.0 ± 0.3	6.8 ± 0.7
1	143.3 ± 31.7	63.0 ± 0.5	53.9 ± 1.6	7.0 ± 0.9	5.1 ± 0.7	174.5 ± 42.5	77.2 ± 9.9	69.9 ± 16.4	7.8 ± 1.3	6.0 ± 0.5
5	147.3 ± 15.8	61.5 ± 1.9	54.4 ± 1.2	7.6 ± 0.5	4.7 ± 0.6	154.1 ± 27.3	71.2 ± 14.6	62.0 ± 8.9	7.4 ± 1.2	5.8 ± 0.9
10	146.1 ± 19.7	62.6 ± 3.2	54.0 ± 1.5	7.5 ± 0.6	4.8 ± 0.1	166.5 ± 27.0	77.0 ± 10.7	65.0 ± 5.7	7.7 ± 1.0	5.6 ± 0.4
20	141.2 ± 20.0	63.2 ± 3.5	54.1 ± 1.5	7.5 ± 1.2	4.7 ± 0.7	157.7 ± 31.1	81.3 ± 14.1	69.3 ± 9.6	7.9 ± 0.8	5.0 ± 0.5

T_V_: maximum temperature in the vicinity of the vessel, T_C_: maximum temperature on the contralateral side of the vessel, d_V_: penetration depth on the vessel side, d_C_: penetration depth on the contralateral side of the vessel.

**Figure 3:**
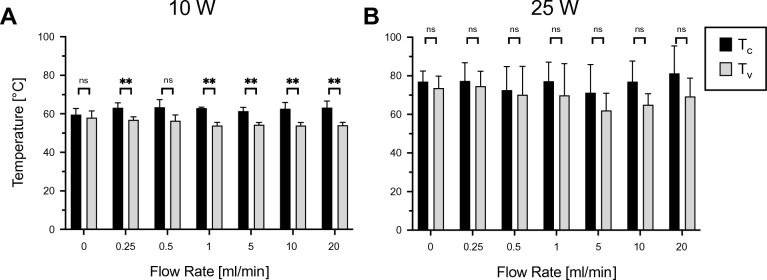
Temperature analysis. Maximum temperatures at different flow rates for 10 W (**A**) and 25 W (**B**). T_V_: thermocouple in the vicinity of the vessel, T_C_: thermocouple on the non-perfused side of the ablation zone (control). *p < 0.05, **p < 0.01, ns *p* > 0.05.

At a constant power delivery of 10 W, a significant temperature decrease between T_C_ and T_V_ was observed with increasing flow rates. T_V_ decreased from 58.1 ± 3.4 °C (0 ml/min) to 54.1 ± 1.5 °C (20 ml/min) (Fig. 3A).

T_C_ and T_V_ were higher when applying a power level setting of 25 W compared to 10 W with T_V_ ranging from 69.3 ± 9.6 °C (20 mL/min) to 73.7 ± 6.1 °C (0 ml/min) and reaching temperatures up to 81.3 ± 14.1 °C (20 ml/min) on the control side (T_C_) (Fig. 3B).

T_V_ was consistently below T_C_ at both power levels 10 W and 25 W and across all flow rates. Significant differences between T_C_ and T_V_ were found at a power level setting of 10 W for flow rates of 0.25, 0.5, 1, 5, 10 and 20 ml/min. At a power level setting of 25 W, the tendency towards lower T_V_ values though systematically observed, did not meet the level of statistical significance for the flow rates examined.

Regression analysis was performed to establish a relationship between the flow rate (ln(ml/min)) and the measured temperatures (°C). At a power level setting of 10 W, T_V_ showed a significant decrease with rising flow, indicating a flow rate dependency of the observed heat sink effect. On the non-perfused contralateral side T_C_ was not significantly impacted by changing flow rates. At a power level setting of 25 W, an analogous flow rate dependency of T_V_ was observed, whilst not statistically significant (Fig. 5 A,B).

### Ablation zone analysis

**Figure 4:**
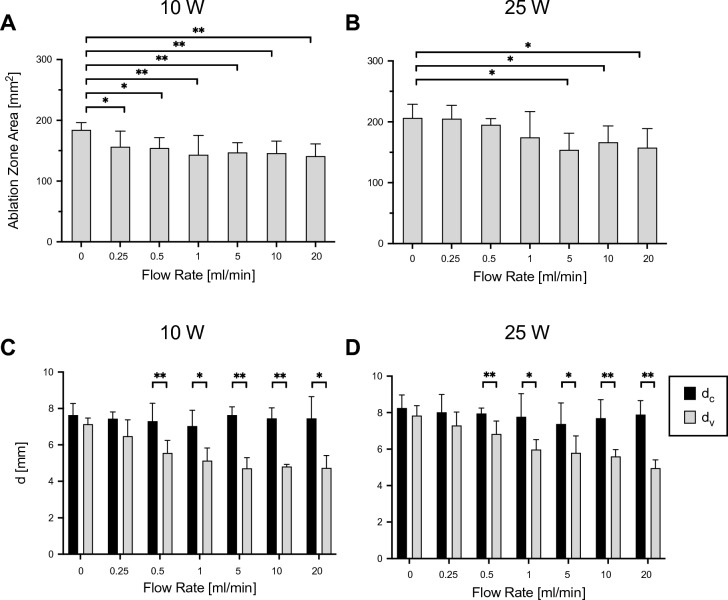
Cross-section analysis. Ablation zone area at different flow rates at 10 W (**A**) and 25 W (**B**). Penetration depth (d) on both the vessel and contralateral side at different flow rates with power levels set to 10 W (**C**) and 25 W (**D**). *p < 0.05, **p < 0.01.

At a power level of 10 W, the ablation zone area decreased from 184 ± 12 mm^2^ (0 ml/min) to 141 ± 20 mm^2^ (20 ml/min) (Fig. 4). When applying the 25 W power level setting, the total ablation zone area reached a maximum of 207 ± 22 mm^2^ (0 ml/min), decreasing to 158 ± 31 mm^2^ (20 ml/min) with increasing flow rates (Fig. 3B).

At constant 10 W power delivery, all flow rates from 0.25 to 20 ml/min showed a significant decrease in cross-sectional area compared to the control group. In the 25 W group, a significant difference compared to the control was found at flow rates of 5, 10 and 20 ml/min.

**Figure 5:**
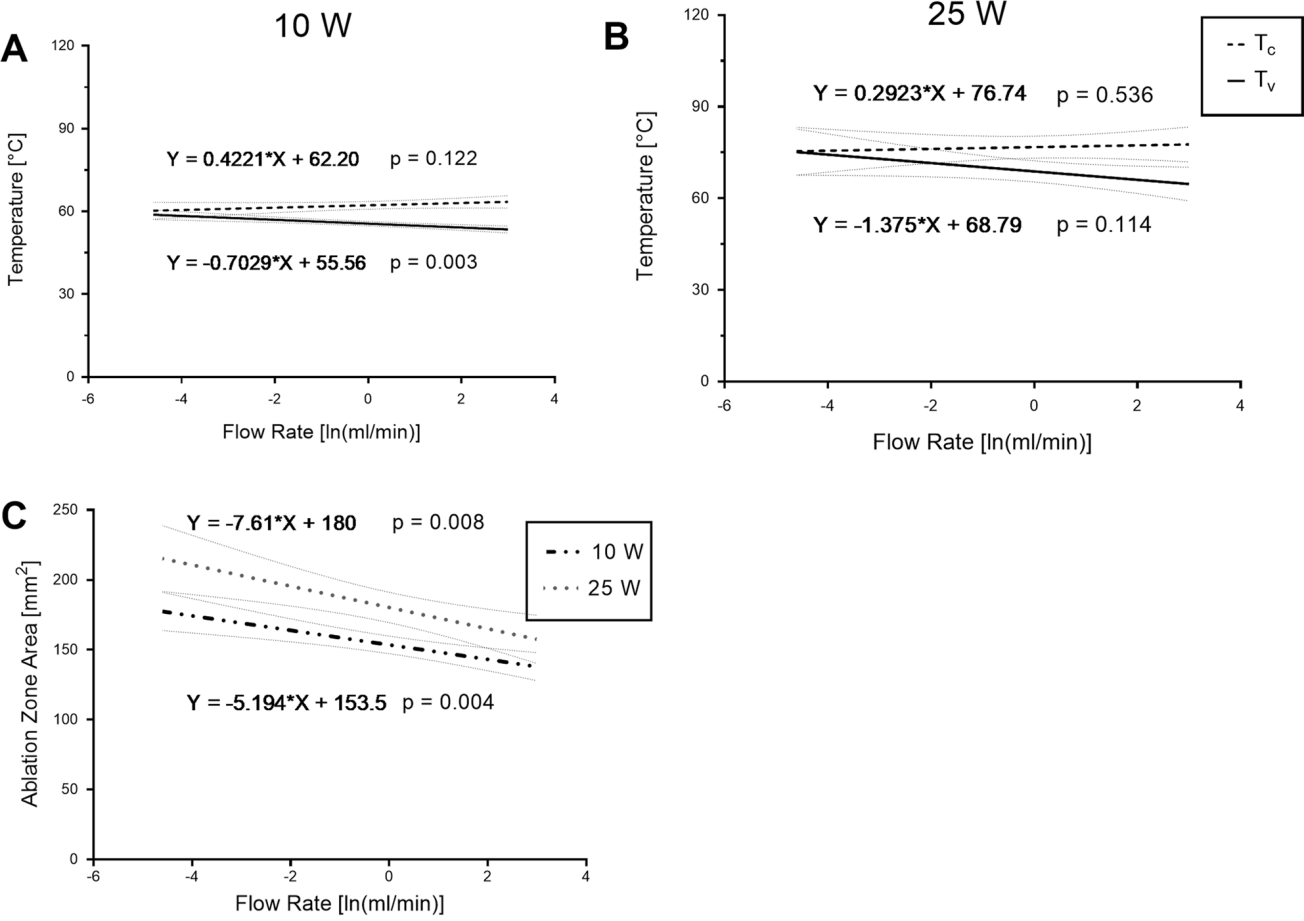
Regression analysis of flow rate impact on temperature and ablation zone area at power level settings of 10 W and 25 W. Trendline of temperature on the vessel side and the contralateral as a function of flow rate at a power level setting of 10 W (**A**) and 25 W (**B**). (**C**) relationship between flow rate and the ablation zone area at both power level settings of 10 W and 25 W.

At each flow rate and power setting, the ablation zone tissue penetration depth d_V_ on the vessel side was decreased compared to the penetration depth on the control side d_C_ (Fig. 4C,D). The mean ablation zone penetration depth d_C_ remained stable around 7.4 mm with increasing flow rates, while d_V_ decreased from 7.1 ± 0.3 (0 ml/min) to 4.7 ± 0.7 mm (20 ml/min) in the 10 W series. In the 25 W group, the mean ablation zone penetration depth d_C_ reached around 8.0 mm. With increasing flow rates, d_V_ showed a decrease from 7.8 ± 0.5 (0 ml/min) to 5.0 ± 0.5 mm (20 ml/min). Significant differences in the mean ablation zone penetration depth between d_C_ and d_V_ at both power level settings of 10 W and 25 W were observed at all flow rates from 0.5 to 20 ml/min. The total ablation zone area showed a significant negative association with the flow rate (ln(ml/min)) at both power level settings of 10 W and 25 W, suggesting that the heat sink effect was present regardless of the chosen power level setting (Fig. 5C).

## Discussion

The heat sink effect, a phenomenon observed in the proximity of perfused blood vessels during thermal ablation procedures, poses a potential obstacle to ablation efficacy as previously shown in liver-based thermal ablation models^11,29^. To examine this effect in the context of thyroid ablation, an ex vivo experimental setup was established aiming to mimic thyroid-specific ablation conditions. Using bipolar RFA in bovine thyroid tissue samples, significant heat sink phenomena were observed, impacting both ablation zone temperature and subsequent ablation zone expansion. Effects attributable to heat sink were seen at low flow rates (≤ 1 ml/min) delivered to the ablation zone through a fine glass tube (3 mm outer diameter) which served as an analogue for directional intrathyroidal blood flow.

Based on previous experimental findings, 60 °C is commonly considered the temperature threshold for successful induction of instantaneous thermal damage to targeted tissues^28,30^. In our study, at a power level setting of 10 W, the cooling effect of the perfused vessel induced a temperature decrease below 60 °C in the vicinity of the vessel, while on the contralateral side no significant decrease was observed. The decrease in the total ablation zone area was primarily attributable to a significant reduction of the ablation zone expansion on the vessel side, likely caused by lower ablation zone temperatures and interruption of conductive heat propagation. The reduced tissue penetration on the vessel side suggests that the decrease in ablation zone area is caused by the directional heat sink effect, particularly noticeable in the periphery of the perfused vessel adjacent to the ablation probe.

Ringe et al. investigated heat sink effects in the context of hepatic microwave ablation^31^. Their findings demonstrated a significant reduction of the ablation zone area in a porcine liver model in correlation with increasing flow rates. The maximum measured temperatures in the center of the ablation zone ranged from around 40–80 °C depending on the applicator-vessel distance (10–20 mm). The observed flow rate-dependent impact on the ablation zone area is generally in line with observations from our study. However, the flow rates used in their study were specific to the liver, set to 700 and 1400 ml/min, thus with limited applicability to the thyroid gland.

Lehmann et al. examined the heat sink effect in an ex vivo liver model using bipolar RFA with liver-specific settings^32^. Their study found significant heat sink in liver tissue, even at flow rates as low as 1 ml/min. Our results confirm the existence of thermal decrease exerted by a small glass vessel (outer diameter of 3 mm) at comparably low flow rates. Moreover, it could be shown that even at lower flow rates down to 0.25 ml/min, quantifiable, directional cooling effects on the surrounding thyroid tissue are detectable. The observed slight tendency towards lower temperatures on the vessel side even in the absence of active flow (0 ml/min), suggests that the mere presence of a water filled vessel may itself contribute to temperature decline by absorbing and dissipating heat from the surrounding tissue.

In our model, a vessel diameter of 3 mm was sufficient to induce heat sink effects within the ablation zone. This vessel caliber was chosen based on expected diameters of main thyroidal blood vessels^33^, and following the rationale that smaller vessels would likely be subject to thermal damage and thus less relevant in limiting ablation success. In vivo research by Yu et al. showed the role of vessel diameter in a study using microwave ablation in a porcine liver model. Their histological analysis demonstrated that vessel size significantly affected the extent of heat sink, with lesions extending to the vein wall in 39–56% of cases involving vessels larger than 3 mm, compared to 78% when the vessel diameter was less than 3 mm^29^.

Overall, the investigation revealed a larger ablation zone area when ablation was performed at a higher maximum power level up to 25 W compared to continuous delivery of 10 W (206.6 ± 22.4 vs. 184.2 ± 11.9 mm in the control group). These findings compare well with a wide range of studies performed in ex vivo liver models, where increased power input translates into larger ablation zone expansion^34^. Our results show a relationship between the flow rate and both the temperature on the vessel side and the cross-sectional ablation zone area in both power setting groups. Yet, in the 25 W power level group, the activation of resistance-adapted power delivery, which continuously adjusts power output based on tissue resistance, likely contributed to an increased variability of measurements impacting the levels of statistical significance upon intragroup comparison. Based on findings from regression analysis and the measured tissue penetration depth, a relevant flow rate-dependent heat sink can still be assumed also in the 25 W group.

The experimental setup used in this study relies on an established concept allowing for controlled and repeatable investigation of a single bipolar RFA shot under the influence of constant perfusion. However, limitations to this model must be pointed out. First, there are intrinsic limits to the presented ex vivo approach, which lacks the possibilty of simulating the effects of microperfusion and vasomotor response that may be present in a living organism. Second, the use of a glass tube to simulate the perfused blood vessel, which cannot fully match the properties of biologic tissue in terms of elasticity, dynamic responsiveness, and heat-absorbing capacity. Yet, the applicability of glass tubes as substitutes for vascular flow has been previously established and discussed in several studies^25,35^. Third, the setup used involved a fixed position of the vessel placed in parallel to the applicator at a distance of 2 mm. While this vessel-to-applicator relation allowed for controlled investigation of vascular flow at a potentially critical position within the ablation zone, it cannot account for the wide range of scenarios encountered in clinical practice, where orientation and vessel-to-applicator distance can vary considerably.

Results from our study may have relevant clinical implications. So far, it has been unclear whether previous findings on heat sink phenomena observed in liver tissue are also applicable to thyroid ablation. In thyroid RFA, clinical studies have identified the peripheral zone of benign nodules and its frequently prominent vasculature as a potential risk factor for late nodule regrowth^36^. In their study evaluating the impact of unablated thyroid tissue on long-term outcomes, Lim et al. reported regrowth rates in the range of 5–9% during a follow-up period of up to 4 years^37^. Residual viable thyroid tissue after RFA as a potential source of regrowth has been shown to be more likely located at the peripheral margin of treated nodules, as identified by Sim et al.^38^. To optimize homogeneous heat delivery and long-term outcomes in thyroid RFA, the concept of vascular targeting has been proposed as a promising approach to mitigate the heat sink effect, as evidenced by the results of a recent study^39^. Using vascular-directed ablation techniques should be considered especially relevant in the treatment of AFTNs or proposed treatment concepts for papillary microcarcinomas^9,40^, where full coverage of the target tissue with sufficient levels of energy is an imperative for successful and safe treatment.

## Conclusion

Flow rate-dependent heat sink effects, even at low flow rates ≤ 1 ml/min, were observed in an ex vivo model using thyroid-specific ablation conditions. In thyroid nodules with prominent vasculature, heat dissipation through perfusion may thus result in clinically relevant limitations to ablation efficacy and should be considered for treatment planning.

### Supplementary Information

Supplementary Figure S1.

## Data Availability

The datasets used and/or analyzed during the current study are available from the corresponding author on reasonable request.

## References

[1] Kim J (2018). 2017 thyroid radiofrequency ablation guideline: Korean society of thyroid radiology. Korean J. Radiol..

[2] Dobnig H (2019). Radiofrequency ablation of thyroid nodules: “Good clinical practice recommendations” for Austria. Wien Med. Wochenschr..

[3] Papini E (2019). Minimally-invasive treatments for benign thyroid nodules: A Delphi-based consensus statement from the Italian minimally-invasive treatments of the thyroid (MITT) group. Int. J. Hyperther..

[4] Feldkamp J (2020). Non-surgical and non-radioiodine techniques for ablation of benign thyroid nodules: Consensus statement and recommendation *. Exp. Clin. Endocr. Diab..

[5] Papini E, Monpeyssen H, Frasoldati A, Hegedüs L (2020). 2020 European thyroid association clinical practice guideline for the use of image-guided ablation in benign thyroid nodules. Eur. Thyroid J..

[6] Orloff LA (2022). Radiofrequency ablation and related ultrasound-guided ablation technologies for treatment of benign and malignant thyroid disease: An international multidisciplinary consensus statement of the American Head and Neck Society Endocrine Surgery Section with the Asia Pacific Society of Thyroid Surgery, Associazione Medici Endocrinologi, British Association of Endocrine and Thyroid Surgeons, European Thyroid Association, Italian Society of Endocrine Surgery Units, Korean Society of Thyroid Radiology, Latin American Thyroid Society, and Thyroid Nodules Therapies Association. Head Neck.

[7] Sung JY (2015). Radiofrequency ablation for autonomously functioning thyroid nodules: A multicenter study. Thyroid.

[8] Tong M (2019). Efficacy and safety of radiofrequency, microwave and laser ablation for treating papillary thyroid microcarcinoma: A systematic review and meta-analysis. Int. J. Hyperth..

[9] Mauri G (2021). European Thyroid Association and Cardiovascular and Interventional Radiological Society of Europe 2021 clinical practice guideline for the use of minimally invasive treatments in malignant thyroid lesions. Eur. Thyroid J..

[10] Organ LW (1976). Electrophysiologic principles of radiofrequency lesion making. Stereot. Funct. Neurosurg..

[11] Pregel P (2021). Radiofrequency thermoablation on ex vivo animal tissues: Changes on isolated swine thyroids. Front Endocrinol..

[12] Nikfarjam M, Muralidharan V, Christophi C (2005). Mechanisms of focal heat destruction of liver tumors. J. Surg. Res..

[13] Kohlhase KD (2016). Bipolar radiofrequency ablation of benign thyroid nodules using a multiple overlapping shot technique in a 3-month follow-up. Int. J. Hyperth..

[14] Li X (2016). Treatment efficacy and safety of ultrasound-guided percutaneous bipolar radiofrequency ablation for benign thyroid nodules. Br. J. Radiol..

[15] Dobnig H, Amrein K (2019). Value of monopolar and bipolar radiofrequency ablation for the treatment of benign thyroid nodules. Best Pract. Res. Cl. En..

[16] Mulier S (2005). Local recurrence after hepatic radiofrequency coagulation. Ann. Surg..

[17] Goldberg SN (1998). Percutaneous radiofrequency tissue ablation: Does perfusion-mediated tissue cooling limit coagulation necrosis?. J. Vasc. Interv. Radiol..

[18] Lehmann KS (2016). Minimal vascular flows cause strong heat sink effects in hepatic radiofrequency ablation ex vivo. J. Hepato-Bil-Pan Sci..

[19] Poch FGM (2016). The vascular cooling effect in hepatic multipolar radiofrequency ablation leads to incomplete ablation ex vivo. Int. J. Hyperth..

[20] Pillai K (2015). Heat sink effect on tumor ablation characteristics as observed in monopolar radiofrequency, bipolar radiofrequency, and microwave, using ex vivo calf liver model. Medicine.

[21] Zhao C-K (2016). Factors associated with initial incomplete ablation for benign thyroid nodules after radiofrequency ablation: First results of CEUS evaluation. Clin. Hemorheol. Micro.

[22] Sim JS, Baek JH (2021). Long-term outcomes of thermal ablation for benign thyroid nodules: The issue of regrowth. Int. J. Endocrinol..

[23] Sim JS, Baek JH (2021). Unresolved clinical issues in thermal ablation of benign thyroid nodules: Regrowth at long-term follow-up. Korean J. Radiol..

[24] Cho SJ, Baek JH, Chung SR, Choi YJ, Lee JH (2019). Thermal ablation for small papillary thyroid cancer: A systematic review. Thyroid.

[25] Lehmann KS (2009). Ex situ quantification of the cooling effect of liver vessels on radiofrequency ablation. Langenbeck’s Arch. Surg..

[26] Welp C, Siebers S, Ermert H, Werner J (2006). Investigation of the influence of blood flow rate on large vessel cooling in hepatic radiofrequency ablation/Untersuchung des Einflusses der Blutflussgeschwindigkeit auf die Gefäßkühlung bei der Radiofrequenzablation von Lebertumoren. Biomedizinische Technik. Biomed. Eng..

[27] Mulier S (2007). Experimental and clinical radiofrequency ablation: Proposal for standardized description of coagulation size and geometry. Ann. Surg. Oncol..

[28] Thomsen S (1991). Pathologic analysis of photothermal and photomechanical effects of laser–tissue interactions. Photochem. Photobiol..

[29] Yu NC (2008). Microwave liver ablation: Influence of hepatic vein size on heat-sink effect in a porcine model. J. Vasc. Interv. Radiol..

[30] Zervas NT, Kuwayama A (1972). Pathological characteristics of experimental thermal lesions: Comparison of induction heating and radiofrequency electrocoagulation. J. Neurosurg..

[31] Ringe KI (2015). Experimental evaluation of the heat sink effect in hepatic microwave ablation. PLoS ONE.

[32] Lehmann KS (2016). Minimal vascular flows cause strong heat sink effects in hepatic radiofrequency ablation ex vivo: Minimal vascular flows cause strong heat sink effects in hepatic radiofrequency ablation ex vivo. J. Hepato-Bil-Pan Sci..

[33] Ozgur Z, Govsa F, Celik S, Ozgur T (2008). Clinically relevant variations of the superior thyroid artery: An anatomic guide for surgical neck dissection. Surg. Radiol. Anat..

[34] Goldberg SN, Gazelle GS, Solbiati L, Rittman WJ, Mueller PR (1996). Radiofrequency tissue ablation: Increased lesion diameter with a perfusion electrode. Acad. Radiol..

[35] Poch FGM (2022). Cooling effects occur in hepatic microwave ablation at low vascular flow rates and in close proximity to liver vessels—ex vivo. Surg. Innov..

[36] Ahn HS, Kim SJ, Park SH, Seo M (2016). Radiofrequency ablation of benign thyroid nodules: Evaluation of the treatment efficacy using ultrasonography. Ultrasonography.

[37] Lim HK (2013). Radiofrequency ablation of benign non-functioning thyroid nodules: 4-year follow-up results for 111 patients. Eur. Radiol..

[38] Sim JS, Baek JH, Lee J, Cho W, Jung SI (2017). Radiofrequency ablation of benign thyroid nodules: depicting early sign of regrowth by calculating vital volume. Int. J. Hyperth..

[39] Offi C (2021). The ablation of thyroid nodule’s afferent arteries before radiofrequency ablation: Preliminary data. Front. Endocrinol..

[40] Cesareo R (2020). Radiofrequency ablation on autonomously functioning thyroid nodules: A critical appraisal and review of the literature. Front. Endocrinol..

