# Supplementary material for: Heat sink effects in thyroid bipolar radiofrequency ablation: an ex vivo study
Source: Sci Rep. 2023 Nov 7;13:19288. doi: 10.1038/s41598-023-45926-2 (PMC10630443; doi:10.1038/s41598-023-45926-2)

Heat Sink Effects in Thyroid Bipolar Radiofrequency Ablation: An Ex Vivo Study

Konrad Klimek[1], Nicolai Mader[1], Christian Happel[1], Amir Sabet[1], Frank Grünwald[1], Daniel Groener[1]*

[1]Goethe University, Department of Nuclear Medicine, University Hospital Frankfurt, Germany

Konrad Klimek
Department of Nuclear Medicine, University Hospital, Goethe University, Frankfurt
Email: konrad.klimek@kgu.de

Dr. Nicolai Mader
Department of Nuclear Medicine, University Hospital, Goethe University, Frankfurt
Email: nicolai.mader@kgu.de

Dr. Christian Happel, MSc
Department of Nuclear Medicine, University Hospital, Goethe University, Frankfurt
Email: christian.happel@kgu.de

PD Dr. Amir Sabet, MSc
Department of Nuclear Medicine, University Hospital, Goethe University, Frankfurt
Email: amir.sabet@kgu.de

Prof. Dr. Frank Grünwald
Department of Nuclear Medicine, University Hospital, Goethe University, Frankfurt
Email:frank.gruenwald@kgu.de

Dr. Daniel Groener
Department of Nuclear Medicine, University Hospital, Goethe University, Frankfurt
Email: groener@med.uni-frankfurt.de

Corresponding author: Daniel Groener
Department of Nuclear Medicine
University Hospital Frankfurt
Theodor Stern Kai 7
60590 Frankfurt, Germany
Tel.: +49 (0) 69 6301 6803
Fax: +49 (0) 69 6301 6805
E-mail: groener@med.uni-frankfurt.de

KEYWORDS:

Heat sink, thyroid, bipolar radiofrequency ablation, ex vivo model

SUPPLEMENT

Figure S1:

(A) Top view illustration of the custom-built polycarbonate cutting device. The positioning device was designed to ensure consistent and precise cuts through the central cross-section of the ablation zone.

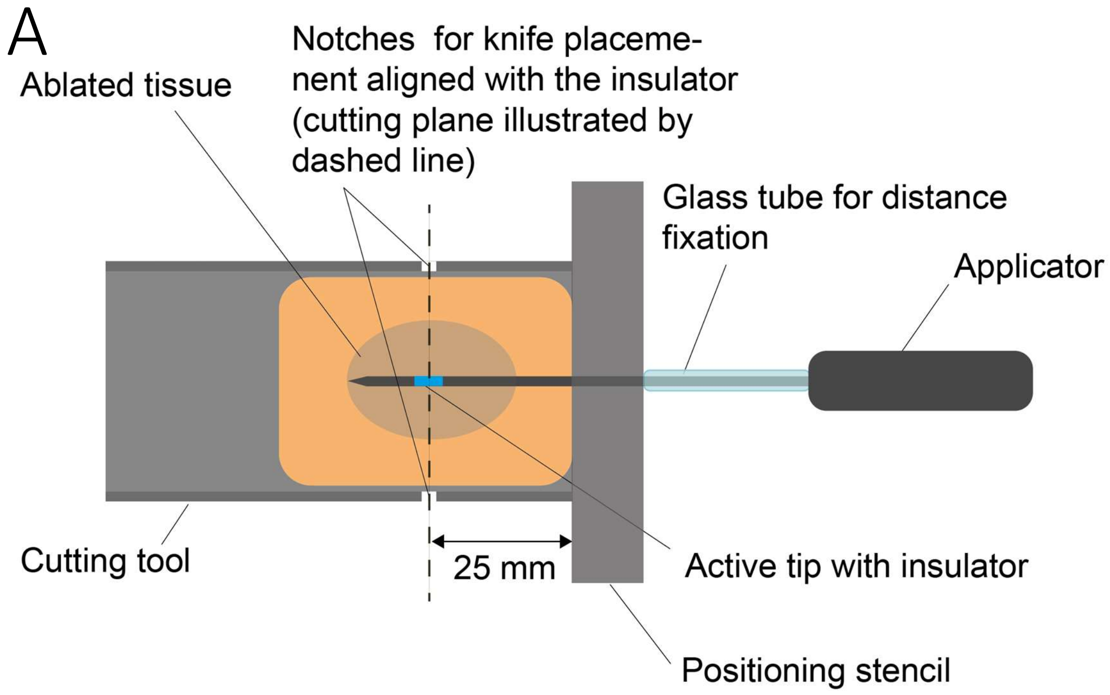

(B) Overhead photograph of the cutting tool. To ensure repeatable cross-sectioning, the cutting knife is introduced through the notches that align with the ablation probe’s insulator (plane with the largest diameter of the ablation cross-section). The device keeps the tissue probe in position during the cutting procedure.

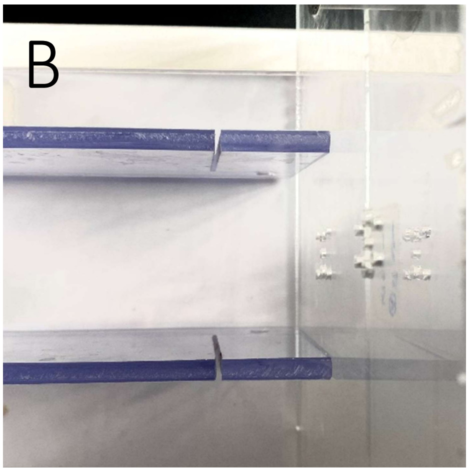

Supplement: Supplementary file 1 — Supplementary Figure S1. [file 41598_2023_45926_MOESM1_ESM.pdf]
